# Recurrent Remitting Seronegative Symmetrical Synovitis With Pitting Edema in a Nonagenarian: An Atypical Clinical Course Requiring Methotrexate for Remission

**DOI:** 10.1002/jgf2.70098

**Published:** 2026-01-07

**Authors:** Ken Sato

**Affiliations:** ^1^ Toyota Municipal Okabayashi Clinic Toyota Aichi Japan

## Abstract

Remitting seronegative symmetrical synovitis with pitting edema (RS3PE) syndrome is a rare inflammatory arthritis of older adults. We report a 92‐year‐old man who presented with fatigue and distal pitting edema, fulfilling the clinical features of RS3PE. Although corticosteroids are typically effective, he showed an incomplete response and relapsed despite dose escalation. Addition of methotrexate resulted in complete remission, with normalization of inflammatory markers. Elderly‐onset rheumatoid arthritis and paraneoplastic RS3PE were considered but were clinically unlikely. This case highlights that RS3PE can occur even in nonagenarians and may require disease‐modifying therapy when corticosteroid responsiveness is insufficient.

## 1 Background

Remitting seronegative symmetrical synovitis with pitting edema (RS3PE) syndrome is a rare inflammatory arthritis occurring in older adults, first described by McCarty in 1985. Its characteristic features include acute‐onset symmetrical synovitis, prominent pitting edema of the distal extremities, seronegativity for rheumatoid factor (RF) and anti–cyclic citrullinated peptide antibodies (ACPA), absence of radiographic erosions, and a generally good response to low‐dose corticosteroids [1].

Because hand involvement and systemic symptoms such as fatigue are common, RS3PE can significantly impair quality of life. Although early diagnosis and timely treatment are essential, identification can be challenging because of nonspecific symptoms unless characteristic physical findings are recognized. The exact pathophysiology of RS3PE syndrome remains unclear; however, elevated levels of vascular endothelial growth factor, a mediator of increased vascular permeability, have been proposed to contribute to synovitis and distal pitting edema [2].

We report an atypical case of RS3PE syndrome in a man in his nineties who relapsed during corticosteroid therapy and ultimately achieved remission following the addition of methotrexate (MTX).

## 2 Case Presentation

**FIGURE 1:**
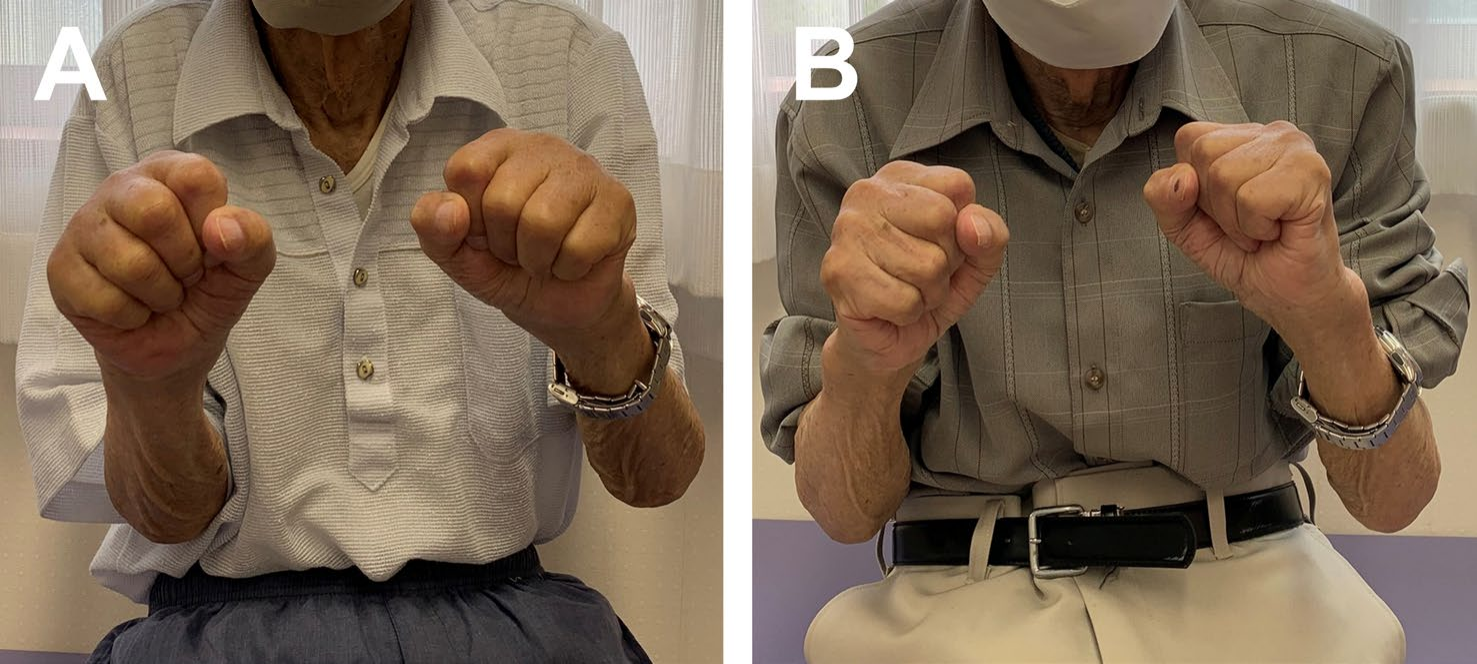
(A) “Boxing‐glove”–like swelling of the dorsal hands at initial presentation. (B) Complete resolution of dorsal hands swelling after 1 month of MTX therapy.

A 92‐year‐old man with a history of hypertension and benign prostatic hyperplasia presented during a routine visit with fatigue and anorexia that had developed abruptly over 3–4 days and persisted for 1 month. On physical examination, he exhibited bilateral pitting edema of the dorsum of the hands and feet (Figure 1A). The dorsal hands were tender and warm, with pain exacerbated by grip. No abnormalities were noted in the large joints, and he was afebrile.

Laboratory testing showed an erythrocyte sedimentation rate of 95 mm/h (reference 1–7 mm/h), C‐reactive protein (CRP) 9.72 mg/dL (reference < 0.30 mg/dL), and matrix metalloproteinase‐3 (MMP‐3) 2541.5 ng/mL (reference 36.9–121.0 ng/mL). Tests for RF, ACPA, and antinuclear antibody were negative. Tumor markers—including carcinoembryonic antigen, carbohydrate antigen 19–9, alpha‐fetoprotein, and prostate‐specific antigen—were within normal limits. Hand radiographs revealed no erosions, and musculoskeletal ultrasonography demonstrated subcutaneous edema and tenosynovitis of both flexor and extensor tendons. No findings suggested heart failure, hypothyroidism, deep vein thrombosis, or drug‐induced edema. Based on these findings, RS3PE syndrome was diagnosed, and oral prednisolone (PSL) 10 mg/day was initiated.

After 2 weeks of treatment, fatigue and anorexia persisted, prompting an increase in PSL to 15 mg/day. Two weeks after dose escalation, systemic symptoms resolved, dorsal hand edema improved, and the CRP level decreased to 2.3 mg/dL. The same dose was continued. One month later, fatigue worsened again, with recurrence of edema of the hands and feet and CRP elevation to 11 mg/dL. MTX 4 mg/week was added. After 1 month of combination therapy, systemic symptoms and peripheral edema resolved completely (Figure 1B), and CRP normalized to 0.3 mg/dL. PSL was gradually tapered, and MTX was increased to 6 mg/week for maintenance. The patient has since remained in remission for 6 months of follow‐up without relapse. No adverse events related to methotrexate or corticosteroid therapy were observed during follow‐up.

## 3 Discussion

We encountered a case of RS3PE syndrome occurring in a nonagenarian, which ultimately required MTX to achieve remission because of an incomplete response to corticosteroids. First, RS3PE can occur even in the very elderly, including nonagenarians. Second, although RS3PE typically shows a rapid and favorable response to corticosteroids, some patients may exhibit poor steroid responsiveness, in which case MTX can be a useful therapeutic option.

The onset of RS3PE in nonagenarians has been described only rarely [1]. Nevertheless, as illustrated by our patient, RS3PE can present in this age group, sometimes with only nonspecific symptoms such as anorexia or general fatigue. In our case, careful physical examination was crucial for identifying the characteristic pitting edema of the distal extremities, which enabled the early diagnosis of RS3PE.

Regarding the atypical treatment course, the first consideration was whether the diagnosis was appropriate. Although RS3PE syndrome typically improves rapidly with corticosteroids [1, 3], our patient relapsed during therapy and achieved remission only after MTX was introduced. His clinical manifestations and laboratory findings were compatible with RS3PE, with incomplete corticosteroid responsiveness as the only atypical feature. Important differential diagnoses include elderly‐onset rheumatoid arthritis (EORA) and paraneoplastic RS3PE syndrome. EORA is more common in women, generally has a more gradual onset, and predominantly involves proximal joints [4]. Imaging studies often show synovial hypertrophy or bone erosions [5], which were not observed in our patient. Paraneoplastic RS3PE, in contrast, is more common in men, frequently presents with systemic symptoms [6], and is characterized by markedly elevated inflammatory markers and MMP‐3 levels [7]. Such cases are also known for poor steroid responsiveness and frequent relapse [6, 7]. Although our patient shared some of these features, initial evaluations found no evidence of malignancy, and tumor marker levels were normal. Therefore, idiopathic RS3PE remains the most appropriate diagnosis at this time. Nevertheless, given reports that malignancy may be detected during the course of RS3PE even when not evident at diagnosis, long‐term follow‐up remains essential, particularly in patients with atypical or relapsing disease courses.

Atypical disease courses in RS3PE have been reported. While most cases respond promptly to corticosteroids, a minority experience relapse or insufficient remission [1]. Origuchi et al. reported that approximately 10% of patients required disease‐modifying antirheumatic drugs within 1 year of diagnosis [8].

## 4 Conclusion

This case demonstrates that RS3PE syndrome can occur even in the very elderly and may relapse during corticosteroid therapy. To preserve activities of daily living and quality of life, early remission is essential. When response to corticosteroid therapy is insufficient, MTX can be a reasonable option, with careful differentiation from EORA and paraneoplastic RS3PE.

## Author Contributions

**Ken Sato:** conceptualization, data curation, formal analysis, investigation, methodology, project administration, writing – original draft.

## Funding

The author has nothing to report.

## Consent

This case was conducted in accordance with the declaration of Helsinki. Written informed consent was obtained from the patient for publication of this case report.

## Conflicts of Interest

The author declares no conflicts of interest.

## Data Availability

Data sharing not applicable to this article as no datasets were generated or analysed during the current study.
